# Surface coordination chemistry of MXenes: defects, terminations and anchored metal sites for inorganic redox catalysis

**DOI:** 10.3389/fchem.2026.1908009

**Published:** 2026-07-28

**Authors:** Haotian Wu, Shengjun Ji, Wenyue Si, Yinxu Xie, Jiawen Tian

**Affiliations:** College of Mechanical Engineering, Shandong Huayu University of Technology, Dezhou, Shandong, China

**Keywords:** defect engineering, electrocatalysis, inorganic chemistry, MXene, single-atom catalyst, surface termination

## Abstract

MXenes are usually reviewed as a broad family of two-dimensional carbides and nitrides, yet their most distinctive inorganic-chemistry feature is a dense, reactive and electronically coupled surface. This mini review frames MXenes as configurable coordination platforms rather than passive conductive supports. We discuss how terminal groups, metal vacancies, heteroatom substitution, interlayer ions, reconstruction and single-atom anchoring define local metal-ligand environments that regulate adsorption, charge transfer and catalytic selectivity. Representative examples from hydrogen evolution, oxygen electrocatalysis, single-atom-catalyst-mediated small-molecule redox chemistry, and rechargeable metal–air or lithium–oxygen chemistry are integrated with foundational studies on synthesis and surface characterization. The central message is that progress depends less on adding more components to MXene hybrids than on specifying which inorganic site is responsible for the reaction and how that site evolves under operating potentials.

## 1 Introduction

MXenes, commonly written as M_n+1_X_n_T_x_, occupy a distinctive position in inorganic materials chemistry because a transition-metal carbide or nitride lattice is exposed through a monolayer-scale surface populated by O, OH, F, Cl or related terminal ligands. The discovery and development of Ti_3_C_2_T_x_ and related compositions established MXenes as processable two-dimensional solids, but a more chemically useful description is that each sheet behaves as an extended coordination object whose metal centers are only partly saturated by carbon or nitrogen and partly saturated by external ligands (Alhabeb et al., 2017; Halim et al., 2016; Hong Ng et al., 2017; Naguib et al., 2021; Xu and Gogotsi, 2020). This view connects synthesis, surface analysis, and catalysis: etching removes the A layer from a MAX phase, delamination opens interlayer space, and the resulting surface terminations decide how electrons, ions and adsorbates couple to the transition-metal sublattice (Alhabeb et al., 2017; Ghidiu et al., 2016; Seredych et al., 2019; Tang et al., 2022).

From a mechanistic perspective, the key question is not simply which MXene gives the highest activity, but which local coordination environment is created and how that environment affects adsorption, charge transfer and active-state evolution. Early work on ion exchange, cation solvation and charge storage showed that Ti_3_C_2_T_x_ is not an inert conductor; protons, alkali cations and solvent molecules reorganize near the surface and alter redox accessibility (Ghidiu et al., 2016; Okubo et al., 2018; Tang et al., 2018). Later theoretical and experimental studies demonstrated that terminations and intercalants tune work function, conductivity and optical response, while solid solutions and multilayer transition-metal arrangements add a second level of metal-site control (Deysher et al., 2019; Han et al., 2020; Hart et al., 2019; Schultz et al., 2019). These observations indicate that MXene catalysis should be discussed in terms of site families: terminally ligated basal-plane metals, edge and vacancy metals, substituted lattice metals, intercalated cation fields, reconstructed oxyhydroxide domains and externally anchored atoms or clusters.

At the same time, this chemically addressable surface is intrinsically vulnerable to oxidation and reconstruction, especially under anodic potentials, alkaline electrolytes and oxygen-evolving conditions (Seredych et al., 2019). Therefore, the coordination environment discussed in this review should not be regarded as a static structural label. It may correspond to an as-synthesized precursor, a resting state after electrolyte exposure, or a reconstructed turnover state under catalytic potentials (Razzaq et al., 2024). This instability also defines the evidentiary requirement for MXene catalysis. *Ex situ* XPS shifts, impedance changes or improved electrochemical activity can suggest a coordination effect, but they rarely prove the identity of a turnover site on their own. Operando or *in situ* methods, including Raman spectroscopy, X-ray absorption spectroscopy, infrared spectroscopy and potential-dependent surface analysis, are therefore essential for connecting a designed surface motif to a working active state (Che Mohamad et al., 2025). When such methods are unavailable, post-reaction characterization, leaching tests, poisoning experiments and controlled reference catalysts should be used to distinguish experimentally supported assignments from plausible but still speculative interpretations.

This review advances that site-centered framework. The first part outlines how synthetic choices define the ligand inventory and electronic baseline of MXenes. The second part evaluates five inorganic coordination motifs that recur across recent redox-catalysis studies. The third part considers how these motifs behave under reaction conditions, because a site observed in an *ex situ* spectrum may be only a precursor to the active state. Throughout, recent research examples are embedded into this framework rather than treated as isolated reports. The scope is deliberately bounded: MXene composites have many uses in sensing, batteries, membranes and electronics, but the focus here is surface coordination chemistry relevant to redox electrocatalysis and coupled ion-electron transfer (Ajmal et al., 2025; Fei et al., 2025; Lim et al., 2020; Morales-García et al., 2020; Wu X. et al., 2024).

This framing further explains why MXenes remain attractive despite the large number of alternative two-dimensional materials. Graphene, transition-metal dichalcogenides and layered oxides can provide high surface area, but MXenes combine metallic or near-metallic transport with chemically addressable transition-metal sites. The same sheet can host ion intercalation, surface ligand exchange, lattice substitution and external metal anchoring. Accordingly, the surface resembles a dense array of inorganic coordination fragments rather than a chemically uniform plane. For readers in inorganic and catalytic chemistry, this distinction matters because it shifts attention from morphology descriptors such as nanosheet size or restacking toward coordination descriptors that can be compared across reactions: ligand identity, metal oxidation state, vacancy density, interlayer ion population and reconstruction product.

Another reason for adopting a coordination framework is that MXene literature spans both foundational reviews and rapidly expanding application reports. Broad reviews have documented MXene discovery, processing and energy-storage behavior (Naguib et al., 2021; Okubo et al., 2018; Tang et al., 2018; Xu and Gogotsi, 2020), while recent catalysis-focused reviews have summarized MXene electrocatalysts for water splitting, surface modulation, heterostructure interfaces and heterogeneous catalytic performance (Bhat et al., 2024; Lim et al., 2020; Liu et al., 2020; Morales-García et al., 2020; Sajid et al., 2024; Thalji et al., 2025; Tian et al., 2026; Wu Y. et al., 2024; Zhang et al., 2025). Although these summaries are valuable, a mini review must add a sharper organizing idea. Here, the organizing idea is that every synthetic or operational modification should be translated into a local inorganic site description. When this translation is possible, different studies become comparable. When it is not possible, performance trends should be treated as empirical rather than mechanistic. Building on these recent reviews, the present mini review does not attempt to re-catalogue MXene catalytic systems, but instead evaluates how convincingly surface terminations, defects, dopants, interlayer species, anchored atoms and reconstructed interfaces can be assigned to specific coordination environments and active states.

Unlike previous reviews that mainly classify MXene catalysts by reaction type, composite architecture or performance metric, this mini review organizes the field by surface coordination motifs and uses these motifs as mechanistic variables rather than descriptive labels. The intended contribution of this framework is not to rename existing categories such as terminations, defects, dopants or anchored active sites, but to ask how these structural features participate in active-site assignment. In a conventional performance-centered review, a Ti_3_C_2_T_x_ hybrid, a doped carbide and a reconstructed oxide/MXene interface may be compared mainly by overpotential, current density or durability. In the surface-coordination framework used here, these systems are compared by whether a defined metal–ligand environment can be linked to a specific adsorbed intermediate, charge-transfer process or reconstructed turnover state. This distinction enables three mechanistic questions to be asked across different reactions: whether the proposed site is experimentally observed, whether it remains stable or reconstructs under operating potentials, and whether the catalytic trend can be separated from conductivity, wetting, surface area or mass-transport effects. Therefore, the central aim of this review is not to catalogue high-performance MXene catalysts, but to evaluate how convincingly a specific surface coordination environment can be assigned to a specific reaction intermediate, electron-transfer process or active state.

**FIGURE 1:**
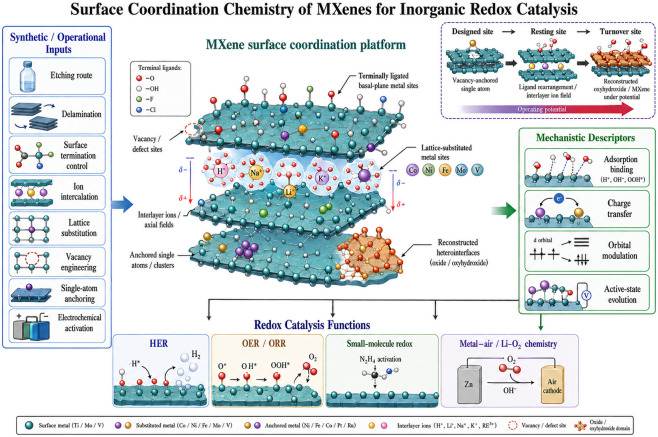
Surface-coordination framework for MXene inorganic redox catalysis. The schematic integrates MXene synthesis, local coordination microenvironments, mechanistic descriptors and redox functions. Etching and surface modification convert MAX precursors into terminated M_n+1_X_n_T_x_ MXene sheets, while intercalation, lattice substitution, vacancy formation, anchored single atoms and reconstructed interfaces define local active sites. These sites regulate adsorption, charge transfer, orbital modulation and active-state evolution, linking MXene surface chemistry to HER, OER/ORR, small-molecule redox catalysis, and metal–air or Li–O_2_ chemistry.

The term “coordination” is used here in a broad but concrete sense. It includes the primary bonds between a surface transition metal and C or N atoms in the MXene lattice, the terminal ligand bonds to O, OH, F or Cl, secondary-sphere interactions with interlayer ions and solvent, and metal-metal or metal-support interactions at anchored atoms and clusters. These interactions are not equally strong, yet each can alter the free energy of an adsorbed intermediate. In that sense, MXenes bridge molecular coordination chemistry and extended solid-state chemistry: the local site has ligand-field character, while the extended sheet supplies conductivity, mechanical integrity and a tunable electrostatic environment. Figure 1 summarizes the review’s central framework: MXene synthesis and operation generate local surface coordination environments that connect mechanistic descriptors with redox-catalysis functions.

## 2 Synthetic control of the ligand field

The first coordination variable is surface termination chemistry. Fluoride-containing etchants, *in situ* HF routes, molten salts and Lewis-acidic methods generate different mixtures of O, OH, F, Cl and vacancy-like sites; these groups are not decorative species but ligands that change the electron density and steric accessibility of surface metals (Alhabeb et al., 2017; Naguib et al., 2021; Seredych et al., 2019; Tang et al., 2022). Thermal analysis and high-temperature studies indicate that terminations can be removed, transformed or reorganized, producing surfaces that are chemically distinct from the as-synthesized solid (Seredych et al., 2019). Work-function measurements on Ti_3_C_2_T_x_ further show that the same carbide backbone can behave as a different electron donor or acceptor depending on termination identity and coverage (Hart et al., 2019; Schultz et al., 2019). For catalysis, this means a reported activity trend cannot be interpreted from nominal composition alone.

MXene coordination environments can be constructed through several synthetic routes. Conventional HF or *in situ* HF etching of MAX phases is widely used to remove the A layer and introduce mixed –O/–OH/–F terminations, but this route often couples delamination, defect formation and termination chemistry in a single step (Alhabeb et al., 2017; Naguib et al., 2021). Lewis-acidic molten-salt and related halide routes broaden the precursor space beyond conventional Al-containing MAX phases and can generate Cl-rich or other halogen-containing surfaces, thereby providing different control over surface ligands and oxidation state (Li et al., 2019; Li et al., 2020; Lu et al., 2019; Shen et al., 2021). Post-synthetic strategies, including ion intercalation, thermal or electrochemical activation, ligand exchange and metal anchoring, can further tune interlayer chemistry, termination distribution, vacancy density, reconstructed domains and single-atom coordination (Ghidiu et al., 2016; Kamysbayev et al., 2020; Razzaq et al., 2024; Zhou et al., 2022). For catalytic interpretation, these approaches should be compared not only by yield, morphology or flake size, but also by the type of coordination site they generate and by how stable that site is under reaction conditions.

The term “ligand field” is used here as a mechanistic analogy rather than as a claim that practical MXene surfaces possess a single, molecularly defined coordination geometry. Real MXene catalysts usually contain mixed O/OH/F/Cl terminations, adsorbed water, interlayer ions, edge sites, partial oxidation products and local defects. Therefore, the surface coordination environment should be understood as a statistical distribution of local metal–ligand motifs rather than a uniform ligand field. This limitation is important because averaged formulas such as Ti_3_C_2_T_x_ or averaged XPS-derived termination ratios cannot by themselves identify the specific site that binds H*, OH*, OOH* or oxygenated intermediates.

Intercalation introduces a second layer of inorganic control. Ion-exchange studies show that cations and their solvation shells occupy the interlayer galleries and modify charge storage kinetics; the cation therefore becomes part of the local reaction environment rather than merely a spectator electrolyte (Ghidiu et al., 2016; Okubo et al., 2018). Recent thin-film and ellipsometric studies of Ti_3_C_2_T_x_ with intercalated species reinforce this point by linking interlayer chemistry with broadband optical and electronic response (Furchner et al., 2024). In catalytic films, intercalants can change sheet spacing, electrolyte access, local pH and the electric field experienced by adsorbed intermediates. This helps to explain why nominally similar MXene catalysts can differ when prepared with different delamination agents, washing histories or supporting electrolytes.

The metal sublattice provides a third design variable. Mo-based MXenes, Mo-V layered compositions and solid-solution MXenes demonstrate that the transition-metal layer can be chemically diversified before or during etching (Deysher et al., 2019; Halim et al., 2016; Han et al., 2020). This diversification matters because adsorbate binding on a carbide surface is governed by both surface ligands and the d-band character of the underlying metals. Single-site Co substitution in two-dimensional molybdenum carbide, for example, enhanced hydrogen-evolution activity by introducing isolated metal centers into a carbide matrix rather than by simply increasing surface area (Kuznetsov et al., 2019). This result foreshadowed the current interest in vacancy-rich MXenes and anchored single atoms, where the support supplies a conductive, ligand-rich scaffold and the active metal supplies a defined orbital set (Bai and Guan, 2023; Zhou et al., 2022).

The catalytic effect of a surface functionality depends not only on its chemical identity but also on the MXene backbone on which it is located. Surface terminations such as –O, –OH, –F and –Cl can change local hydrophilicity, metal–ligand bonding, work function, interlayer accessibility and adsorbate binding, but these effects depend strongly on termination coverage and spatial heterogeneity (Hart et al., 2019; Schultz et al., 2019; Tang et al., 2022). O-rich surfaces may strengthen metal–oxygen interactions and stabilize OH*, OOH* or other oxygenated intermediates, whereas F-rich or Cl-rich surfaces may block adsorption sites, alter work function or modify ion accessibility depending on coverage. OH-containing surfaces and interlayer water can participate in hydrogen bonding, cation solvation and proton-coupled interfacial processes, but their role may vary with electrolyte pH and hydration state (Ghidiu et al., 2016). Similarly, the same termination can have different catalytic consequences on Ti-, V-, Mo-, Nb- or mixed-metal MXenes because the M-site electronic structure, carbide/nitride bonding, conductivity and oxidation tendency differ. Mo-based MXenes and Ti/Nb/V-based solid-solution MXenes illustrate how changing the metal sublattice can modify the electronic baseline before surface chemistry is considered (Deysher et al., 2019; Halim et al., 2016; Han et al., 2020). Therefore, surface functionality and MXene type should be discussed together rather than treated as independent descriptors.

Synthetic control remains imperfect. Many MXene studies report T_x_ as a collective label, but catalysis requires a more explicit chemical description: What fraction of O, OH, F or Cl is present? Are vacancies present before reaction or generated electrochemically? Does the metal atom sit in a carbon/nitrogen vacancy, bind to surface O, or reside at an edge? Recent Raman, vibrational and surface-imaging studies are beginning to answer these questions by resolving degradation pathways, sublattice reactivity and even atomic-scale surface manipulation (Sumaiya et al., 2022; Yoo et al., 2025). The field will become more mechanistically predictive when these methods are paired with electrochemical measurements rather than used only as post-synthesis characterization.

The synthetic history of an MXene film can also influence catalysis through mesoscale effects that can be mistaken for coordination effects. Delamination can increase accessible area, but it can also remove or exchange interlayer cations, change flake edges and modify oxidation susceptibility. Guidelines for Ti_3_C_2_T_x_ processing therefore remain mechanistically relevant even when a paper is focused on catalysis rather than synthesis (Alhabeb et al., 2017). A catalyst ink prepared from a freshly delaminated colloid is chemically different from one made from an aged powder, and a film dried with trapped solvent may expose a different interlayer environment than a vacuum-annealed film. These variables should be reported because they influence the ligand field and transport field at the same time.

A further synthetic challenge is the coexistence of intended and unintended phases. Oxidizing MXenes can generate metal oxides on disordered carbon sheets, a transformation that can be useful for hybrid catalysts but can also obscure whether the active phase is a carbide, an oxide or an interface between them. Early oxidation studies and later stability analyses demonstrate that such transformations are common rather than exceptional. Therefore, when an MXene-derived oxide or sulfide composite is highly active, the mechanistic claim should specify whether MXene chemistry is responsible for precursor templating, electronic coupling, defect generation, or active-site formation. This distinction is particularly important for heterostructures in which the added component is itself catalytically competent (Bhat et al., 2024; Lim et al., 2020; Zhao et al., 2024).

The ideal synthesis section of a future MXene catalysis paper would therefore read less like a procedural recipe and more like a coordination-site construction. It would state why a particular etchant is chosen, which ligands it is expected to introduce, which defects it may create, and how subsequent washing or intercalation changes the coordination environment. It would also distinguish deliberate dopants from impurities. This matters for single-atom and trace-metal studies because very small amounts of Pt, Ni, Co or Fe can dominate catalytic response if they occupy the right vacancy or edge site. Quantitative methods for trace single atoms are thus not peripheral quality-control tools; they are necessary for assigning inorganic active sites.

## 3 Families of active sites on MXenes

Five conceptual site families are used here to organize the most frequently discussed inorganic mechanisms in MXene-based redox catalysis. These families are not intended to be exhaustive, mutually exclusive or structurally isolated. Real MXene catalysts also contain edge sites, grain boundaries, mixed-termination domains, restacked interlayer regions and partially oxidized patches. These motifs may dominate reactivity when basal planes are blocked, when flakes are small, or when electrochemical activation preferentially attacks edges and defect-rich domains. Therefore, the five-site classification should be read as an organizing framework for mechanistic discussion rather than as a complete map of all possible active sites.

The first family comprises terminated basal-plane metal sites. Here, a surface metal is coordinated to carbide or nitride atoms below and terminal ligands above. O-rich terminations often strengthen hydrophilic interactions and can stabilize oxygenated intermediates, whereas F-rich surfaces may lower conductivity or block adsorption depending on coverage (Schultz et al., 2019; Tang et al., 2022; Zhang et al., 2025). The second family comprises vacancy or defect sites. Removing a surface metal or generating a carbon/nitrogen-related defect creates undercoordinated atoms and stronger anchoring sites for incoming metals. Vacancy-rich MXenes immobilizing Ni single atoms for hydrazine oxidation illustrates how a defect can transform a conductive sheet into a single-site catalyst platform (Zhou et al., 2022).

The third family involves lattice substitution. Replacing one transition metal with another inside the MXene lattice can tune adsorption without creating a separate phase. Co-substituted Mo_2_C MXene for hydrogen evolution is an early example, and more recent solid-solution MXenes or multimetal oxide/MXene nanoarchitectures extend related design logic by coupling multiple metal d states with conductive MXene frameworks (Han et al., 2020; He et al., 2026; Kuznetsov et al., 2019). The fourth involves interlayer or axial coordination fields. Intercalated rare-earth ions, transition metals or organic or metal-organic ligands can impose local electrostatic fields and axial coordination motifs that reshape d-orbital occupation (Fatima et al., 2025; Tran et al., 2025). The fifth comprises reconstructed interfaces, in which MXene partly transforms into oxides, oxyhydroxides, TiN-like domains or supported heterostructures under synthesis or operation (Che Mohamad et al., 2025; Kaplan et al., 2025; Wu X. et al., 2024; Zhao et al., 2024).

Table 1 summarizes these site families, their synthetic handles, primary inorganic variables and evidence required for validated active-site assignment. Rather than ranking catalysts by overpotential, the table emphasizes how an observed catalytic response can be connected to a defined MXene-related coordination environment. This distinction is important because many MXene hybrids contain several plausible active motifs at once, including the parent MXene surface, a deposited oxide or sulfide, edge defects and a reconstructed layer. Without explicit site assignment, the phrase “MXene-enhanced catalysis” risks becoming descriptive rather than explanatory.

**TABLE 1:** Surface coordination motifs and key evidence for assigning MXene-related active sites in inorganic redox catalysis.

Site family	Synthetic handle	Primary inorganic variable	Evidence required for validated assignment	Assignment risk
Terminated basal-plane metal sites	Etching route; post-treatment; thermal or electrochemical ligand exchange	O/OH/F/Cl identity, coverage and work function	XPS or high-resolution XPS for termination composition and metal oxidation states; Raman/IR or NMR for surface groups; work-function measurements; DFT adsorption energies for H*, OH* or OOH*; correlation between termination variation and HER/OER activity (Bai et al., 2021; Hart et al., 2019; Schultz et al., 2019; Tang et al., 2022; Zhang et al., 2025)	T_x_ is often averaged; mixed terminations, adsorbed water and operation-induced ligand exchange may hide the actual active ligand
Vacancy and undercoordinated sites	Vacancy-rich synthesis; electrochemical activation; defect-preserving delamination	Metal coordination number, vacancy density and exposed carbide/nitride environment	EPR or positron annihilation for defect identification; aberration-corrected STEM for missing atoms or defect-rich regions; XAS/EXAFS for coordination number; vacancy-density/activity correlation; poisoning or leaching tests for defect-anchored sites (Sumaiya et al., 2022; Yoo et al., 2025; Zhou et al., 2022)	Vacancies may heal, oxidize or bind adventitious species; defect signals may overlap with edge sites, oxidation products or trace-metal impurities
Lattice-substituted metal sites	MAX precursor design; solid-solution or multimetal MXene synthesis	Dopant identity, d-state distribution and local metal-carbon/nitrogen coordination	STEM-EDS/EELS mapping for dopant distribution; XAS/EXAFS for local coordination; XRD/Rietveld or phase analysis for solid-solution formation; comparison with physical mixtures or secondary-phase controls; DFT analysis of intermediate adsorption (Han et al., 2020; He et al., 2026; Kuznetsov et al., 2019)	Bulk composition may not represent exposed surface sites; dopants may segregate, oxidize or form secondary phases
Interlayer or axial coordination fields	Cation exchange; rare-earth or transition-metal intercalation; axial organic or metal-organic ligands	Interlayer ion identity, local electric field, solvation structure and axial ligand strength	XRD d-spacing analysis; XPS/XAS or ICP for intercalant identity and loading; *in situ* Raman/IR for ion or ligand evolution; electrochemical ion-exchange tests; DFT or ligand-field analysis of O_2_/OOH*/O_2_^−^ binding (Fatima et al., 2025; Furchner et al., 2024; Ghidiu et al., 2016; Liu et al., 2025; Tran et al., 2025)	Intercalants or axial ligands may migrate, dissolve or exchange with electrolyte ions; activity may arise from spacing, wetting or conductivity changes rather than coordination-field effects
Reconstructed heterointerfaces	Operando oxidation; anodic activation; oxide/oxyhydroxide/sulfide coupling; MXene-derived precursor transformation	Residual carbide/oxide coupling, metal-OH/metal-O/metal-OOH motifs and active-state evolution	Operando Raman or XAS for reconstruction; post-reaction TEM/XPS/XAS; time-dependent activity and surface-composition tracking; leaching tests; comparison among fresh, conditioned and deliberately oxidized catalysts (Che Mohamad et al., 2025; Kaplan et al., 2025; Razzaq et al., 2024; Wu et al., 2024a; Wu et al., 2024b)	The starting MXene may be only a precatalyst; the active site may be a reconstructed oxide/oxyhydroxide or residual carbide/oxide interface rather than pristine MXene

To avoid overinterpreting performance trends, this review distinguishes three levels of mechanistic support. A validated assignment requires direct or operando evidence for the proposed coordination site under relevant reaction conditions, together with appropriate control catalysts and stability or leaching analysis. A probable assignment is supported by consistent *ex situ* spectroscopy, electrochemical trends, adsorption-energy calculations and comparison with related systems, but lacks direct observation of the turnover site. A speculative assignment is inferred mainly from activity enhancement, impedance reduction or post-reaction surface shifts without sufficient controls. This distinction is used below to separate experimentally supported active-site assignments from plausible but still unverified interpretations.

The overlap among these families is mechanistically important rather than problematic when the study is designed to isolate variables. A vacancy can anchor a single atom; an intercalant can alter the field around a reconstructed oxide; a substituted lattice metal can change the oxidation pathway of the surface. This overlap is an advantage if the study is designed to isolate variables, but it becomes problematic when all changes are introduced simultaneously. For example, a molten-salt route may alter termination chemistry, introduce defects, produce a heterostructure and change flake morphology in one step. A convincing mechanism would then require a control series that separates these effects, such as a defect-rich MXene without the heterostructure, the heterostructure on a non-MXene support and the same MXene after passivating the suspected active site.

The same logic helps to interpret single-atom catalysts on MXenes. Single atoms are often presented as isolated metal centers, but their catalytic identity depends on the support ligands. A Ni atom coordinated by vacancy-rich Ti_3_C_2_T_x_ is not equivalent to Ni on nitrogen-doped carbon, and a Pd atom interacting with dual dopants on MXene is not simply a small form of metallic palladium (Huynh et al., 2025; Zhou et al., 2022). The MXene surface can supply axial oxygen ligands, carbide back-bonding, electron donation and geometric confinement. Describing these features explicitly brings MXene single-atom catalysis closer to molecular inorganic chemistry, where coordination number, ligand donor strength and symmetry are central concepts.

For each family, the evidentiary threshold should match the claim. A paper claiming termination-controlled activity should show termination changes and not merely cite a known etching route. A paper claiming vacancy-anchored single atoms should quantify isolated atoms, rule out nanoparticles and connect vacancy density to loading or activity. A paper claiming lattice substitution should demonstrate that the dopant is incorporated into the MXene lattice rather than deposited as a secondary phase. A paper claiming reconstruction should show the precursor and product states. Although these standards may seem demanding, they are the only way to turn MXene catalysis from materials optimization into mechanistic inorganic chemistry.

## 4 Representative redox-catalysis motifs interpreted through site chemistry

Representative MXene-based redox reactions can be more clearly interpreted when the discussion is organized by inorganic site motifs rather than by application categories alone. In this section, four reaction families are emphasized because they directly illustrate how MXene-related coordination environments regulate adsorbed intermediates and electron-transfer steps: hydrogen evolution reaction (HER), oxygen evolution/reduction reactions (OER/ORR), single-atom or small-molecule redox catalysis, and metal–air or lithium–oxygen (Li–O_2_) chemistry. Other energy-storage and optoelectronic applications are not discussed in detail here unless they provide direct evidence for MXene-related active sites, because the central purpose of this review is to define surface coordination chemistry under redox-catalysis conditions rather than to catalogue all MXene-based energy-conversion systems. Recent water-splitting reviews have already summarized MXene material design, surface modulation and HER/OER catalytic performance; therefore, the discussion below focuses on which MXene-related coordination motifs are mechanistically implicated and what evidence is required to assign them as active sites (Thalji et al., 2025).

In hydrogen evolution, MXenes can provide terminally ligated transition-metal sites, substituted carbide environments and electronically coupled metal/MXene interfaces. The relevant inorganic sites include Ti-O, Mo-C, V-C, substituted Mo-C-Co environments and noble-metal clusters coupled to carbide or nitride supports. These sites can regulate H adsorption, water dissociation and electron transfer from the conductive MXene lattice to adsorbed hydrogen-containing intermediates. Co-substituted Mo_2_C MXene is a representative example in which isolated Co sites within a carbide matrix modify hydrogen-evolution activity through local metal-site substitution rather than through a simple surface-area effect (Kuznetsov et al., 2019). MXene-based hybrid architectures and MXene-supported metal catalysts further suggest that metal/MXene interfacial coupling can tune H* binding and charge-transfer pathways (Bai et al., 2021; Sajid et al., 2024; Zhao et al., 2024). For such systems, stronger mechanistic assignment requires termination analysis, oxidation-state characterization, comparison with non-MXene supports and DFT calculations of H*, H_2_O and OH* adsorption. However, many HER reports still infer active sites from lower overpotential, smaller Tafel slope or reduced charge-transfer resistance; such evidence should be treated as probable rather than validated unless paired with termination-resolved spectroscopy, isotope or poisoning controls, non-MXene reference supports or operando analysis.

Oxygen electrocatalysis provides a more dynamic case because the starting MXene surface is often a precatalyst rather than the final turnover site. For OER and ORR-related reactions, the relevant coordination environments may include pristine or partially oxidized V-C, Ti-C and CoFe/MXene interfaces, but the active state can evolve into metal-OH, metal-O and metal-OOH motifs under operating potentials (Che Mohamad et al., 2025; Kaplan et al., 2025; Wu X. et al., 2024; Wu Y. et al., 2024; Zhang et al., 2025). In this context, MXene chemistry contributes not only by improving conductivity or dispersion, but also by providing a reconstructable inorganic precursor that can generate oxyhydroxide-like domains while retaining electronic communication with residual carbide or nitride phases. These reconstructed sites may regulate O-H bond cleavage, OOH* formation, oxygen adsorption and oxygen-release steps. Therefore, mechanistic claims for MXene-based oxygen electrocatalysts should be supported by potential-dependent surface analysis, post-reaction XPS or XAS, operando Raman when available, metal-leaching tests and comparison between fresh and electrochemically conditioned catalysts. Without such evidence, assigning OER or ORR activity to a pristine MXene surface, a specific termination or a reconstructed oxyhydroxide domain should be regarded as speculative, particularly because oxidative potentials can simultaneously change surface composition, metal oxidation state, interlayer chemistry and electrode morphology.

Single-atom and small-molecule redox reactions are especially useful for evaluating MXenes as coordination platforms because selectivity depends strongly on the local metal-ligand environment. Vacancy-rich Ti_3_C_2_T_x_-immobilized Ni single atoms for hydrazine oxidation illustrate this point: the proposed active site is an isolated Ni center stabilized by a vacancy-rich MXene surface, rather than metallic Ni nanoparticles or a generic Ni-containing composite (Zhou et al., 2022). The surrounding carbide and terminal-ligand environment can influence Ni oxidation state, reactant adsorption geometry and N-H activation. Similarly, axially coordinated metal-organic molecules on MXenes show that the MXene surface can act as a secondary coordination environment capable of regulating Fe 3d orbitals and oxygen-related redox steps in rechargeable Zn-air chemistry (Tran et al., 2025). These examples should be interpreted as single-site or axial-field-modified inorganic catalysts, and their site assignments require atomic-resolution imaging, XAS/EXAFS analysis, exclusion of nanoparticles or clusters, poisoning or leaching tests and orbital-level calculations. If isolated atoms, axial ligands or vacancy anchoring are inferred only from increased selectivity or shifted binding energies in calculations, the assignment should be considered probable or speculative until nanoparticle formation, metal aggregation, ligand loss and support-only activity are experimentally excluded.

Metal–air and Li–O_2_ systems extend the same coordination principle to oxygenated intermediates. In these reactions, the important species are not only adsorbed H* or OH*, but O_2_, OOH*, O_2_^−^, peroxide and superoxide intermediates. Axial ligand-regulated single-atom sites in Li–O_2_ batteries demonstrate how metal coordination number, ligand directionality and support geometry can reshape d-orbital occupation and thereby influence oxygen reduction or evolution pathways (Liu et al., 2025). MXene surfaces can also contribute polar O/OH-containing ligands, vacancy-anchored metal centers and interlayer electrostatic fields that stabilize oxygenated intermediates and facilitate charge transfer. However, these systems involve thick electrodes, complex electrolytes and coupled mass transport, so MXene-related coordination effects should be separated from conductivity, wetting and architecture effects. Future reports should combine adsorption-energy calculations, intermediate-binding analysis, post-cycling surface characterization and dissolution or leaching tests to determine whether the MXene component acts as an active coordination platform or mainly as a conductive host. Therefore, mechanisms in metal–air and Li–O_2_ systems should be considered validated only when oxygen-intermediate chemistry, electrode-architecture effects and MXene-derived coordination effects are separated through appropriate controls; otherwise, improved cycling or lower polarization mainly supports a probable performance correlation rather than a confirmed active-site assignment.

To address the need for a comparative discussion of catalytic activities, Table 2 summarizes representative performance metrics of selected MXene-based redox catalysts together with their proposed coordination motifs and evidence status. Because reaction type, electrolyte, catalyst loading, current density, normalization method, electrode architecture and durability protocol differ among studies, these values should not be interpreted as a direct ranking of intrinsic catalytic activity. Instead, the comparison links reported activity metrics with MXene type, surface or coordination motif, mechanistic evidence and evidence level.

**TABLE 2:** Comparative summary of representative catalytic activities of MXene-based redox catalysts and their proposed coordination motifs.

Reaction/system	Representative catalyst	MXene type and coordination motif	Reported catalytic activity	Mechanistic evidence	Evidence status and design message
HER	Mo_2_CT_x_:Co (Kuznetsov et al., 2019)	Mo-based MXene with lattice-substituted Co sites; Co atoms occupy Mo positions in Mo_2_CT_x_	η10 = 0.18 V for Mo_2_CT_x_:Co versus 0.23 V for Mo_2_CT_x_ from background-corrected CV data; onset potential above −70 mV vs. RHE; Tafel slope = 59 mV dec^−1^ for Mo_2_CT_x_:Co versus 78 mV dec^−1^ for Mo2CTx; TOF ≈ 0.1 s^−1^ at −0.25 V vs. RHE	EXAFS confirms isolated Co atoms incorporated into the Mo_2_CT_x_ lattice; control experiment with physical Mo_2_CT_x_ + Co powder showed no activity improvement; DFT attributes improved HER kinetics to modified H binding on O-terminated Mo_2_CO_2_:Co surface	Strong evidence for lattice substitution; probable mechanistic assignment for H* regulation. This example shows that M-site substitution can tune HER activity without forming a separate Co phase
HER/OER/overall water splitting	Te–V_2_C/V_2_O_3_(4:3) (Wu et al., 2024b)	V-based MXene-derived heterointerface with Te doping; self-generated V_2_C/V_2_O_3_ interface	HER η10 = 83.5 mV at −10 mA cm^−2^; HER Tafel slope = 141.1 mV dec^−1^; OER η10 = 279.8 mV at 10 mA cm^−2^; OER Tafel slope = 84.7 mV dec^−1^; overall water-splitting cell voltage = 1.41 V at 10 mA cm^−2^; stable for 24 h under HER and OER tests	H_2_O_2_ microetching generates porous V_2_C/V_2_O_3_ heterointerfaces; Te doping induces local electric-field rearrangement; DFT/DOS analysis shows d-band-center upshift to −0.94 eV and enhanced DOS near the Fermi level, which is linked to improved adsorption/desorption and charge transfer	Probable mechanistic assignment. The data support that controlled oxidation and Te doping convert V_2_C instability into a beneficial heterointerface for bifunctional water splitting
OER	Electro-activated rTi3C2-T (Che Mohamad et al., 2025)	Ti_3_C_2_ MXene with electrochemically induced (oxy)hydroxide-rich reconstructed surface	OER overpotential at 10 mA cm^−2^ ≈ 0.65 V without iR correction; 11% reduction versus untreated rTi_3_C_2_ and 27% reduction versus pristine Ti_3_C_2_; 67% current-density increase at high voltage; Tafel slope = 355 mV dec^−1^ for rTi_3_C_2_-T; apparent activation energy = 13.7 kJ mol^−1^	Operando Raman spectroscopy reveals *in situ* formation of (oxy)hydroxide species; direct intermediate probing and first-principles calculations indicate transition toward the oxide path mechanism; treated rTi_3_C_2_-T shows lower Rct and modified Ti–O/OER-intermediate vibrational signatures	Relatively strong evidence for reconstructed active-state assignment. This example supports the idea that electrochemical surface reconstruction can redirect OER pathways rather than simply degrade MXene
OER/AEM electrolyzer	Co_0.66_Fe_0.34_-LDH@V_1.8_CT_x_, especially CFVv75 (Kaplan et al., 2025)	Vacancy-engineered V_2_CT_x_-derived MXene support with 10% V vacancies; CoFe-LDH/MXene composite	Best OER η10 = 304 mV for CFVv75; best pristine V_2_CT_x_ composite CFV33 gives η10 = 317 mV; 12 h stability at 100 mA cm^−2^ with overpotential increase of 1.0 mV h^−1^ for CFVv75 and 1.4 mV h^−1^ for CFV33; in AEM electrolyzer, CFVv75 gives cell voltage of 1.48 V at 100 mA cm^−2^ and operates 100 mV lower than pure CoFe	*In situ* XAS shows high-valent Co species and irreversible Co coordination changes during OER; post-OER analysis shows Co/Fe are relatively stable but V leaches substantially; vacancy-engineered V_1.8_CT_x_ creates additional accessible sites and different CoFe–MXene interactions	Probable-to-validated active-state evidence, but with important reconstruction/leaching caveat. The example shows that vacancy-engineered V-MXene supports can improve OER and device performance, but the working active state is dynamically reconstructed
Hydrazine oxidation reaction	Ni SACs/Ti_3_C_2_T_x_ (Zhou et al., 2022)	Vacancy-rich Ti_3_C_2_T_x_ MXene immobilizing Ni single atoms; Ni atoms trapped at Ti vacancies and coordinated with C atoms	Onset potential = −0.03 V vs. RHE in 0.1 M hydrazine + 1 M KOH; Tafel slope = 62 mV dec^−1^; Ni loading = 2.93 wt%; only 7.3% current loss after 24,000 s stability test; much lower loss than Ni NPs/Ti_3_C_2_T_x_	HAADF-STEM shows atomically dispersed Ni without nanoparticles; XANES/EXAFS confirms positively charged Ni and Ni–C coordination, with no Ni–Ni feature; DFT shows stronger hydrazine adsorption on Ni SACs/Ti_3_C_2_T_x_ than Ni NPs/Ti_3_C_2_T_x_ and lower total dehydrogenation barrier	Strong evidence for vacancy-anchored single-atom assignment. This example directly supports the concept that MXene vacancies can trap isolated metal atoms and create single-site redox catalysts
Li–O_2_ battery oxygen redox	CoPP-O-MXene (Liu et al., 2025)	O-terminated MXene axially coordinated to Co porphyrin; Co–O axial ligand field modifies Co d orbitals	CoPP-O-MXene LOBs show discharge/charge capacity of 11,035/10,536 mAh g^−1^ and Coulombic efficiency of 95.48%; overpotential = 0.76 V when current density is restored to 100 mA g^−1^; overpotential = 0.86 V at 800 mA g^−1^; cycling stability = 445 cycles	EXAFS/WT-EXAFS indicates axial Co–O coordination and absence of Co–Co bonds; Co coordination number increases from 4.1 in CoPP to 5.4 in CoPP-O-MXene; XPS, *in situ* XRD, DEMS and 3D nano-CT show reversible Li_2_O_2_ formation/decomposition and more uniform Li_2_O_2_ deposition	Strong evidence for axial-coordination-mediated oxygen redox. This example shows that MXene surface oxygen can act as an axial ligand to regulate Co d-orbital occupation and Li_2_O_2_ chemistry

These values should not be interpreted as a direct ranking of intrinsic catalytic activity because reaction type, electrolyte, catalyst loading, electrode architecture, normalization method and durability protocol differ among studies.

Taken together, these representative reaction families and the comparative summary in Table 2 show why MXene catalysis should be discussed through site chemistry rather than through application labels alone. HER highlights hydrogen adsorption and water-dissociation sites; OER and ORR emphasize reconstruction and oxyhydroxide-like active states; small-molecule redox chemistry demonstrates vacancy anchoring and axial coordination; and metal–air or Li–O_2_ chemistry reveals how oxygenated intermediates respond to single-atom sites and secondary coordination fields. This narrowed scope better supports the central thesis of this mini review: MXenes are most valuable for inorganic redox catalysis when their terminal ligands, defects, substituted metals, interlayer ions and reconstructed domains can be assigned to specific reaction intermediates and active-state evolution.

## 5 Stability, reconstruction and the problem of the active state

The most challenging issue in MXene catalysis is that the surface is reactive enough to be useful and unstable enough to change. Oxidation stability studies have shown that Ti_3_C_2_T_x_ and related MXenes can degrade through edge oxidation, basal-plane attack and formation of TiO_2_-like products, depending on water, oxygen, temperature and storage conditions (Bhat et al., 2024; Naguib et al., 2021; Seredych et al., 2019). In electrocatalysis, these pathways can be accelerated or redirected by potential. A catalyst described as “MXene-based” after long operation may contain a reconstructed oxide, oxyhydroxide, nitride, sulfide or carbonaceous residue. This does not invalidate the material; but it means that the active state must be defined operando or at least after controlled electrochemical aging.

Recent evidence for the spontaneous formation of active and selective single-atom centers under anodic polarization is especially important because it suggests that electrochemical potentials can create coordination sites not present in the starting material (Razzaq et al., 2024). In this view, vacancies, terminal O groups and mobile metal atoms are a reservoir from which active sites are assembled during reaction. Surface reconstruction on Ti_3_C_2_ MXene for oxygen evolution likewise indicates that oxyhydroxide-driven pathways can emerge from a carbide precursor (Che Mohamad et al., 2025). These studies shift the design target from static synthesis to programmed transformation: the desired precursor is one that reconstructs into a stable, selective and electronically connected active layer.

Reconstruction should also be treated as a kinetic process rather than only as a thermodynamic endpoint. The rate and extent of MXene transformation can depend on potential window, current density, electrolyte composition, pH, dissolved oxygen, local ion concentration and mass transport within restacked films. For example, a short anodic activation may generate surface oxyhydroxide motifs while preserving electronic coupling to the carbide core, whereas prolonged high-current operation may drive deeper oxidation, metal dissolution or loss of the original interfacial structure. Therefore, reports of reconstructed MXene catalysts should specify activation protocol, electrolyte identity, current density or potential hold, operation time and post-reaction surface depth profile.

Together, these kinetic and structural considerations mean that stability should be discussed at three levels. Chemical stability asks whether the MXene survives storage, washing and ink formulation. Electrochemical stability asks whether the lattice, terminations and anchored species persist at the relevant potential and pH. Mechanistic stability asks whether the assigned active site continues to be the same site during turnover. A catalyst may be chemically unstable but mechanistically useful if it reconstructs reproducibly into the same active phase; conversely, a catalyst may look structurally intact but lose selectivity if intercalants, axial ligands or single atoms migrate. Reports of high activity should therefore be accompanied by post-reaction termination analysis, metal leaching tests, electrochemically active surface normalization and, when possible, operando spectroscopy.

The same stability logic can inform broader MXene applications, but in redox catalysis it is particularly critical because potentials, electrolytes and reactive intermediates can accelerate termination exchange, oxidation, intercalant migration and active-site reconstruction.

Operando terminology should be used carefully. A spectrum collected after polarization is not necessarily operando, and a calculated adsorption energy on a pristine terminated surface may not describe a reconstructed electrode. Nevertheless, combining time-resolved spectroscopy, electrochemical protocols and computation can narrow the possibilities. For oxygen evolution, one can ask whether metal-OOH intermediates bind to a reconstructed oxyhydroxide, an exposed MXene metal, or an anchored single atom. For hydrogen evolution, one can ask whether water dissociation occurs at an oxide/oxyhydroxide domain while hydrogen adsorption occurs at a carbide-coupled metal site. For small-molecule redox reactions, one can ask whether the MXene changes the rate-determining adsorption or electron-transfer step, or merely improves conductivity.

The active-state problem also affects reference selection and comparison across papers. A catalyst reported as Ti_3_C_2_T_x_-supported Pt, V_2_C-derived oxide or CoFe/MXene may have a different active surface after 10 min and after 10 h. Therefore, comparisons should emphasize matched durability, potential range and post-reaction chemistry rather than initial overpotential alone. This is why recent reports on surface reconstruction, MXene-derived architectures and stability pathways are central to the present review even when their immediate applications differ (Che Mohamad et al., 2025; He et al., 2026; Kaplan et al., 2025; Razzaq et al., 2024; Wu X. et al., 2024). They reveal that the inorganic site is a time-dependent object.

A useful way to address this time dependence is to distinguish the designed site, the observed resting site and the turnover site. The designed site is what synthesis intends to create, such as a vacancy-anchored Ni atom or O-terminated Ti center. The resting site is what spectroscopy detects before or after catalysis. The turnover site is the configuration that participates in the rate-determining step. These states may coincide, but often they do not. MXenes make this distinction especially important because ion intercalation, terminal ligand exchange and oxidation can occur on the same timescale as electrochemical conditioning. Mechanistic writing should therefore avoid implying that a pristine structural model automatically represents the working catalyst.

## 6 Design rules and outlook

**FIGURE 2:**
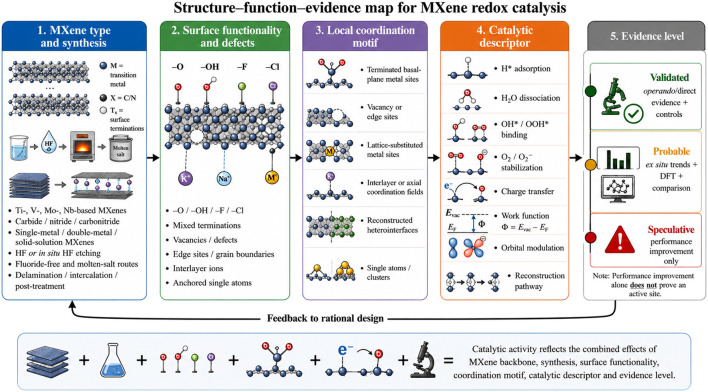
Structure–function–evidence map for MXene redox catalysis. The flowchart summarizes how MXene type, synthetic route and surface functionality jointly determine local coordination motifs and catalytic descriptors. Different MXene backbones, including Ti-, V-, Mo- and Nb-based carbides, nitrides, carbonitrides and solid-solution MXenes, provide different electronic baselines and metal d-state environments. Surface functionalities such as –O, –OH, –F and –Cl, together with vacancies, edge sites, grain boundaries, interlayer ions, reconstructed domains and anchored single atoms, further define local metal–ligand motifs. These motifs regulate H* adsorption, H_2_O dissociation, OH*/OOH* binding, O_2_/O_2_^−^ stabilization, charge transfer, work function, orbital modulation and reconstruction pathways. The final evidence-level block emphasizes that catalytic mechanisms should be interpreted as validated, probable or speculative according to the availability of operando/direct evidence, controlled comparisons and post-reaction verification. The atomic drawings are schematic and are intended to illustrate coordination concepts rather than exact crystallographic models.

Figure 2 summarizes the structure–function–evidence map proposed in this review. It links MXene type, synthetic route and surface functionality to local coordination motifs, catalytic descriptors and evidence levels for active-site assignment. This framework emphasizes that catalytic performance should be interpreted together with coordination-site identity and mechanistic evidence rather than treated as an isolated activity metric. Several design rules therefore emerge from a coordination-centered reading of the MXene literature. First, choose the target active site before choosing the application. If the desired chemistry is hydrogen adsorption, a partially terminated basal-plane metal, substituted Mo site or noble-metal/MXene interface may be appropriate (Bai et al., 2021; Kuznetsov et al., 2019; Zhao et al., 2024). If the desired chemistry is oxygen evolution, a reconstructable metal-oxyhydroxide/MXene interface may be more realistic than an unchanged carbide surface (Che Mohamad et al., 2025; Kaplan et al., 2025; Wu Y. et al., 2024; Zhang et al., 2025). If the desired chemistry requires selectivity among oxygenated intermediates, single atoms or axial ligands on vacancy-rich MXenes offer a more defined coordination environment (Liu et al., 2025; Tran et al., 2025; Zhou et al., 2022).

Second, the ligand field should be reported with the same seriousness as composition. A formula such as Ti_3_C_2_T_x_ is insufficient for mechanistic comparison unless accompanied by termination analysis, interlayer composition, oxidation state and defect information. Surface-termination measurements, vibrational probes, conductive atomic-force microscopy and controlled intercalation experiments should be integrated with electrochemical testing rather than separated into characterization sections (Furchner et al., 2024; Schultz et al., 2019; Sumaiya et al., 2022; Yoo et al., 2025). Third, support effects should be distinguished from site effects. MXenes can improve dispersion, conductivity, hydrophilicity and mass transport, but these advantages do not prove that an MXene metal atom is the active center. Isotope experiments, poisoning tests, operando spectra, density-functional calculations and careful comparison to non-MXene supports are needed when assigning a specific site.

Fourth, reconstruction should be treated as a design variable. Instead of treating oxidation or surface transformation only as failure, researchers can design MXene precursors that form controlled active layers while retaining electronic contact with the carbide or nitride core. This approach aligns with recent work on anodically generated single-atom centers and oxyhydroxide-driven OER pathways (Che Mohamad et al., 2025; Razzaq et al., 2024). Fifth, descriptors should be broadened beyond overpotential. In inorganic chemistry terms, useful descriptors include metal coordination number, ligand identity, vacancy density, axial-field strength, interlayer cation type, work function and reconstruction pathway. These descriptors are closer to cause than performance metrics alone.

Machine learning is becoming increasingly important for MXene research because the compositional and surface-chemical space of MXenes is too large to be explored by experiment or density-functional theory alone. Data-driven models can help predict synthesizability, screen stable MXene compositions, estimate adsorption energies and identify descriptors that connect composition, surface termination and catalytic function. For example, positive and unlabeled machine learning has been used to predict the synthesizability of MXenes and their MAX-phase precursors, providing a way to prioritize experimentally accessible compositions (Frey et al., 2019). Machine-learning models have also been used to predict adsorption energies of gas-phase species on MXene surfaces, which is directly relevant to catalyst design because adsorption energies connect surface composition, termination chemistry and reaction intermediates (Nassar et al., 2025). In addition, deep-learning-enabled high-throughput screening has been used to identify stable MXene photocatalysts for hydrogen production from a large candidate space, showing how ML can reduce the cost of searching compositionally complex MXene families (Tang et al., 2025). Recent reviews of first-principles and machine-learning approaches further emphasize that interpretable descriptors, overfitting control and experimental validation are essential when applying ML to MXene property prediction (Gouveia et al., 2025). These studies suggest that future MXene redox-catalysis research should integrate DFT datasets, experimental synthesis records, surface-termination descriptors and operando-derived active-state information into interpretable models. However, ML predictions should not replace mechanistic validation; predicted active sites or adsorption trends still require synthesis, surface characterization and catalytic testing.

The field is now sufficiently mature to move from cataloguing MXene hybrids to testing falsifiable coordination hypotheses. A strong future study should state a proposed active site, synthesize a precursor designed to generate that site, verify the site before and during reaction, compare it against structurally controlled analogues and report how the site fails. In this sense, future MXene catalysis studies should report not only what performance is improved, but also whether the proposed mechanism is validated, probable or still speculative according to the available site-specific evidence. Such studies would also align MXene catalysis more closely with inorganic and catalytic chemistry by treating MXenes not merely as nanomaterials but as extended inorganic coordination platforms. The practical payoff is clear: by controlling terminal ligands, defects, intercalants and anchored metals together, MXenes can be tuned for selective redox chemistry while retaining the conductivity and processability that made the family attractive in the first place.

A practical reporting checklist could include four blocks. The first is precursor chemistry: MAX phase or alternative precursor, etchant, delamination method, storage time and atmosphere. The second is surface coordination: termination distribution, oxidation state, defect density, interlayer ion identity and any anchored metal loading. The third is catalytic assignment: control supports, poisoning or leaching tests, turnover-normalized activity where possible, and post-reaction or operando evidence for the proposed active site. The fourth is durability: whether the site remains intact, reconstructs into a defined product or dissolves. Such a checklist would make it easier to compare studies that currently use different names for related motifs.

The broader opportunity is to connect MXene chemistry with concepts already familiar in inorganic catalysis. Terminated MXene metals can be compared with surface organometallic fragments; vacancy-anchored atoms can be compared with single-site heterogeneous catalysts; intercalated ions can be compared with secondary-sphere modifiers; reconstructed oxyhydroxide domains can be compared with precatalysts that generate active phases *in situ*. Although these analogies are not perfect, they encourage mechanistic experiments. They also prevent overstatement: an MXene hybrid should not be called a single-site catalyst unless the site distribution and active-state stability support that claim. With this discipline, MXenes can contribute not only high performance but also new inorganic principles for designing reactive two-dimensional surfaces.

In summary, the next step for the field is not simply to discover more MXene composites, but to define more MXene coordination environments. The most compelling studies will connect synthetic variables to a site model, connect the site model to adsorption or electron-transfer evidence, and connect that evidence to a reaction outcome. MXenes are powerful precisely because they are chemically unfinished surfaces: their terminal ligands, defects, interlayers and anchored metals can be adjusted. The challenge is to make these adjustments legible as inorganic chemistry, across synthesis, operation and practical failure analysis under realistic catalytic conditions.
